# Use of IV immunoglobulin to treat steroid resistant, immune checkpoint inhibitor‐induced pure red cell aplasia: A case report

**DOI:** 10.1002/jha2.937

**Published:** 2024-06-16

**Authors:** Sam Sherratt‐Mayhew, Phillip L. R. Nicolson

**Affiliations:** ^1^ Institute of Cardiovascular Sciences University of Birmingham Birmingham UK; ^2^ Department of Haematology University Hospitals Birmingham NHS Foundation Trust Birmingham UK

**Keywords:** immune checkpoint inhibitor, immune‐related adverse events, IV immunoglobulin, pure red cell aplasia

## Abstract

Pure red cell aplasia (PRCA) is characterised by normocytic normochromic anaemia, reticulocytopenia and reduced erythroid precursors in bone marrow. PRCA as an immune‐related adverse event secondary to immune checkpoint inhibitor (ICI) therapy is rare. Steroids are usually used first line to treat ICI‐induced PRCA. Here, we report a case of ICI‐induced PRCA with no response to steroids but where intravenous (IV) immunoglobulin was successfully used second line. ICI therapy was reinitiated following PRCA resolution. PRCA recurrence did not occur.

## 1 INTRODUCTION

Pure red cell aplasia (PRCA) is a rare condition characterised by normocytic normochromic anaemia, reticulocytopenia and reduced erythroid precursors on bone marrow biopsy [1]. PRCA can be congenital but is usually acquired. Acquired PRCA is most commonly idiopathic, but it can be associated with infection, lymphoproliferative disorders or medications such as immune checkpoint inhibitors (ICIs) [1]. Nivolumab and ipilimumab are ICIs inhibiting programmed cell death protein 1 (PD‐1) and cytotoxic T‐lymphocyte‐associated protein 4 (CTLA‐4), respectively. PD‐1 binds to its ligand PD‐L1 and promotes T‐cell apoptosis. PD‐L1 is expressed by many cancer cells to inhibit T‐cell activation [2]. CTLA‐4 outcompetes CD28‐mediated activation of CD80 and CD86, receptors responsible for T‐cell stimulation [3]. Nivolumab and ipilimumab promote T‐cell‐mediated killing of tumour cells and are licensed for the treatment of many malignancies, including uveal and cutaneous melanoma [4]. ICIs are efficacious immunotherapies and do not have the classical side effect profile of cytotoxic agents [5, 6]. Unfortunately, overactivation of the immune system can lead to immune‐related adverse effects against non‐cancerous cells such as in the skin, digestive tract, blood and bone marrow [7]. Immune‐mediated attack of erythroid precursors causes PRCA. Due to the rarity of PRCA, and the relatively novel use of ICIs, few cases of ICI‐related PRCA have been reported [6, 8, 9, 10, 11, 12]. ICI‐induced haematological toxicity can be fatal or can result in significant delays in delivery of anti‐cancer treatments. It is paramount therefore that PRCA is recognised and treated promptly. High‐dose corticosteroids should be used first line, but treatment of steroid‐refractory PRCA is challenging and little literature exists to guide therapy. This report discusses a patient with advanced melanoma who developed steroid‐refractory nivolumab/ipilimumab‐induced PRCA. This responded to intravenous immunoglobulin (IVIG) and the patient was successfully rechallenged with nivolumab.

## 2 CASE REPORT

A 38‐year‐old, white British female with a history of Addison's disease had localised BRAF V600‐mutated melanoma diagnosed 6 years previously. This had been treated with wide local excision. She presented 5 years after excision with local recurrence and distal metastases. She then received dabrafenib and trametinib. Following intolerable side effects, she was switched to nivolumab and ipilimumab.

**FIGURE 1:**
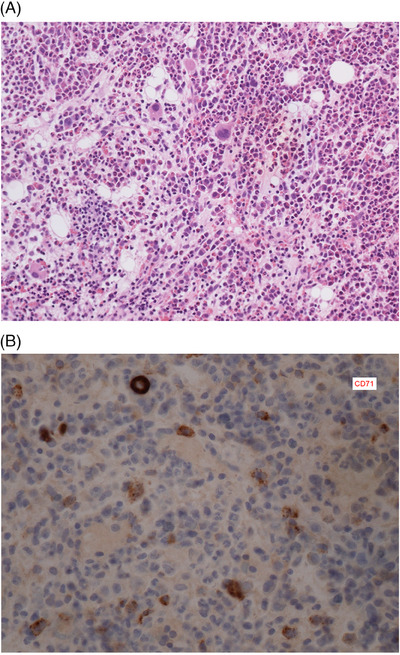
Bone marrow trephine showing marked erythroid hypoplasia with absent erythroid islands when stained with haematoxylin and eosin (A) and markedly reduced erythroid activity when stained with anti‐CD71 (transferrin receptor 1) antibody (B).

Ninety‐seven days after starting nivolumab and ipilimumab, she presented with fatigue and exertional breathlessness. Initial tests found pancytopenia (haemoglobin [Hb] 75 g/L, previously 135 g/L; neutrophils 1.1 × 10^9^/L, previously 2.6 × 10^9^/L; and platelets 66 × 10^9^/L, previously 186 × 10^9^/L; Table 1). Further investigations revealed an undetectable haptoglobin, high lactate dehydrogenase (495 u/L) and a positive direct coombs test (3+) with monospecific testing showing IgG 2+ and C3d 1+, confirming haemolysis. A peripheral blood film did not show any red cell fragments nor any morphological abnormalities of leukocytes or platelets. Reticulocytes were low (5 × 10^9^/L). Viral polymerase chain reaction tests for parvo‐, Epstein–Barr and adenovirus were negative and haematinics were normal. No hepatosplenic imaging was undertaken because recent normal staging cross‐sectional imaging had been performed and there were no abnormal liver function test results. Following these results, a bone marrow aspirate and trephine biopsy showed normal granulo‐ and megakaryopoiesis but absent erythroid islands (Figure 1). This confirmed PRCA mediated through haemolysis of red cell precursors and indicated peripheral consumption of neutrophils and platelets. The temporal association with ICI treatment suggested this as a trigger; daily 1 mg/kg intravenous (IV) methylprednisolone was started and ICIs paused.

**TABLE 1:** Laboratory results.

	Reference range	Before admission	On admission	10 days on steroids	11 days post‐IVIG infusion	31 days post‐IVIG infusion
Hb (g/L)	115–154	135	75	65	120	121
Reticulocytes (×10^9^/L)	33–102	–	5.0	3.7	–	93
WBC (×10^9^/L)	3.0–10.9	4.7	2.79	3.6	8.30	8.34
Platelets (×10^9^/L)	150–400	186	66	130	239	254
Neutrophils (×10^9^/L)	1.5–7.1	2.62	1.10	2.2	6.53	6.59
Lymphocytes (×10^9^/L)	0.6–4.0	1.32	1.10	2.0	0.87	0.79
MCV (fL)	81–102	95.2	90.9	88.8	99.5	99.7
ALT (u/L)	0–55	10	55	18	18	29
AlkP (u/L)	30–130	68	90	75	74	78
Bili (µmol/L)	<21	9	18	11	9	9

Abbreviations: AlkP, alkaline phosphatase; ALT, alanine transaminase; Bili, bilirubin; Hb, haemoglobin; IVIG, intravenous immunoglobulin; MCV, mean cell volume; WBC, white blood count.

After 2 weeks of high‐dose corticosteroids and subsequent wean, neutrophils and platelets normalised but anaemia and reticulocytopenia persisted. Due to the concern of melanoma relapse and a need to restart anti‐cancer treatment, she was then treated with IVIG (1 g/kg, single dose). Following this her fatigue and breathlessness rapidly resolved. A repeat Hb 11 days later was 120 g/L with a reticulocyte count of 93 × 10^9^/L. This remained stable and nivolumab monotherapy was restarted 3 weeks after her IVIG (7 weeks after presentation with PRCA) without PRCA relapse. Unfortunately, her melanoma progressed and she was switched to BRAF targeting treatment with encorafenib and binimetinib before her second nivolumab dose. Six months after her last nivolumab treatment, she remains in remission from PRCA and other cytopenias.

## 3 DISCUSSION

Here, we describe a patient with immune‐mediated PRCA, neutropenia and thrombocytopenia secondary to nivolumab and ipilimumab. The neutropenia and thrombocytopenia were steroid responsive but the PRCA required treatment with IVIG. The PRCA responded rapidly, enabling ICI therapy to restart.

Seventeen cases of ICI‐induced PRCA have been reported, with 15 of these included in a case series from Guo et al. [6, 11, 12]. PRCA was triggered by several different ICIs including nivolumab, pembrolizumab, atezolizumab, durvalumab and ipilimumab, which were paused in all cases.

Of the 17 identified patients, four patients did not receive steroids [8, 12]. Of the 13 patients who were treated with steroids; seven responded but six did not. Of the seven who responded, two suffered from a PRCA relapse upon steroid tapering and needed further treatment with IVIG. Of the six patients who did not respond; three responded to cyclosporin, two were treated successfully with IVIG monotherapy and one had a partial response to IVIG and required further treatment with cyclosporin and steroids to achieve a complete response [8, 9, 10, 11, 12]. Neither of the two treated with IVIG alone suffered from PRCA recurrence and neither were rechallenged with ICI [8, 9].

Like our case, the patient treated with combination therapy was successfully rechallenged with ICI and suffered no PRCA relapse [12]. Unlike our patient however, concurrent treatment with prednisolone, ciclosporin and IVIG was used rather than IVIG monotherapy, making the contribution of IVIG to PRCA remission and prevention of relapse less clear. In both patients there was a need to reinitiate ICI therapy due to likelihood of cancer progression, but this had to be balanced with risk of PRCA relapse. We restarted ICI 3 weeks following IVIG, whereas a delay of 4 months was used by Rueda‐Prada et al. [12]. Of note, while neither patient suffered PRCA relapse, both had melanoma progression after restarting ICI and required a change of anti‐cancer treatment.

Interestingly, a recent cohort study showed increased overall survival in patients with non–small‐cell lung cancer who experienced immune‐related adverse events [13]. It was felt that this was probably due to immune‐related adverse events being correlated with increased immune attack on cancer cells [13]. The cumulative evidence of case reports for ICI‐induced PRCA would agree with this; of the 17 patients reported, eight had favourable cancer outcomes documented, with four unfavourable and five not reported (Supporting Information Table 1).

With worldwide shortages of IVIG, it is important to note that a single dose of 1 g/kg, as used for our patient, can be effective [14]. The mechanism of action of IVIG in treating autoimmune conditions is uncertain. Although multiple mechanisms have been proposed, the most plausible is swamping of Fc receptors on immune cells, leaving them unable to clear the autoantibody‐coated erythroid precursors [15]. Only two of the described patients failed to fully respond to second‐line IVIG, following which further steroids ± ciclosporin was successful in achieving PRCA remission [10, 11, 12]. Whilst the response rates to second‐line ciclosporin are also favourable, the attractiveness of IVIG is the rapid response and the lack of on‐going treatment. We propose that corticosteroids should remain first‐line therapy for ICI‐induced PRCA in most cases, with IVIG used as a treatment of choice for steroid‐refractory disease and that ciclosporin is only started as a third‐line agent. We also provide evidence that it is possible to rechallenge patients with ICI within 4 weeks of IVIG. This is critical in reducing anti‐cancer treatment delays and may thus improve cancer relapse and survival.

## AUTHOR CONTRIBUTIONS

Sam Sherratt‐Mayhew wrote the manuscript. Phillip L. R. Nicolson conceived, reviewed and edited the manuscript.

## CONFLICTS OF INTEREST STATEMENT

The authors declare no conflicts of interest.

## ETHICS STATEMENT

This manuscript complies with the declaration of Helsinki. Patient consent was obtained to share anonymised clinical details. Formal ethical approval for this case report was not required.

## PATIENT CONSENT STATEMENT

The authors have confirmed patient consent statement is not needed for this submission.

## CLINICAL TRIAL REGISTRATION

The authors have confirmed clinical trial registration is not needed for this submission.

## Supporting information

Supporting Information

## Data Availability

Additional anonymised clinical data will be made available upon reasonable request.

## References

[1] Mangla A , Hamad H . Pure red cell aplasia. 2021. [online] PubMed. Available from: https://www.ncbi.nlm.nih.gov/books/NBK549833/. Accessed 24 Jan 2024. 31751023

[2] Shi L , Chen S , Yang L , Li Y . The role of PD‐1 and PD‐L1 in T‐cell immune suppression in patients with hematological malignancies. J Hematol Oncol. 2013;6(1):74. 24283718 10.1186/1756-8722-6-74 PMC3851976

[3] Seidel JA , Otsuka A , Kabashima K . Anti‐PD‐1 and anti‐CTLA‐4 therapies in cancer: mechanisms of action, efficacy, and limitations. Front Oncol. 2018;8:86. 10.3389/fonc.2018.00086 29644214 PMC5883082

[4] Shiravand Y , Khodadadi F , Kashani SMA , Hosseini‐Fard R , Hosseini S. , Sadeghirad H , et al. Immune checkpoint inhibitors in cancer therapy. Curr Oncol. 2022;29(5):3044–3060. 10.3390/curroncol29050247 35621637 PMC9139602

[5] Marin‐Acevedo JA , Kimbrough EO , Lou Y . Next generation of immune checkpoint inhibitors and beyond. J Hematol Oncol. 2021;14(1):45. 10.1186/s13045-021-01056-8 33741032 PMC7977302

[6] Guo Q , Gao J , Guo H , Xie J , Cheng J . Immune checkpoint inhibitor‐induced pure red cell aplasia: case series and large‐scale pharmacovigilance analysis. Int Immunopharm. 2023;114:109490. 10.1016/j.intimp.2022.109490 36459923

[7] Almutairi AR , McBride A , Slack M , Erstad BL , Abraham I . Potential immune related adverse events associated with monotherapy and combination therapy of ipilimumab, nivolumab, and pembrolizumab for advanced melanoma: a systematic review and meta‐analysis. Front Oncol. 2020;10:91. 10.3389/fonc.2020.00091 32117745 PMC7033582

[8] Isoda A , Miyazawa Y , Tahara K , Mihara M , Saito A , Matsumoto M , et al. Pembrolizumab‐induced pure red cell aplasia successfully treated with intravenous immunoglobulin. Int Med. 2020;59(16):2041–2045. 10.2169/internalmedicine.4467-20 PMC7492113 32389947

[9] Nair R , Gheith S , Nair SG . Immunotherapy‐associated hemolytic anemia with pure red‐cell aplasia. N Engl J Med. 2016;374(11):1096–1097. 10.1056/nejmc1509362 26981948

[10] Hall M , Meti N , Liontos L , Cheung MC , Gandhi S . Refractory autoimmune hemolytic anemia and pure red cell aplasia secondary to immunotherapy requiring prolonged immunosuppression. JCO Oncol Pract. 2020;16(10):699–700. 10.1200/OP.20.00047 32603256

[11] Saliba AN , Xie Z , Higgins AS , Andrade‐Gonzalez XA , Fuentes‐Bayne HE , Hampel PJ , et al. Immune‐related hematologic adverse events in the context of immune checkpoint inhibitor therapy. Am J Hematol. 2021;96(10):E362–E367. 10.1002/ajh.26273 34137072

[12] Rueda‐Prada L , Gavrancic T , Cadena‐Sanabria MO , Dumic I , Bourgeois K , King RL , et al. Immune checkpoint inhibitor‐induced pure red cell aplasia: a review of 2 cases in metastatic melanoma. Am J Case Rep. 2023;24:e941789. https://amjcaserep.com/abstract/full/idArt/941789 37957950 10.12659/AJCR.941789 PMC10658056

[13] Cook S , Samuel V , Meyers DE , Stukalin I , Litt I , Sangha R , et al. Immune‐related adverse events and survival among patients with metastatic NSCLC treated with immune checkpoint inhibitors. JAMA Netw Open. 2024;7(1):e2352302. 38236598 10.1001/jamanetworkopen.2023.52302 PMC10797458

[14] So‐Osman C , Delaney M , Fung M , Lu W , Murphy M , Sasongko PL , et al. A global analysis of the use of immunoglobulin, shortages in supply, and mitigating measures: a survey of hospital providers (a BEST Collaborative study). Transfusion. 2024;64:775‐783. 10.1111/trf.17801 38516758

[15] Bayry, J , Misra N , Latry V , Prost F , Delignat S , Lacroix‐Desmazes S , et al. Mechanisms of action of intravenous immunoglobulin in autoimmune and inflammatory diseases. Transfus Clin Biol. 2003;10(3):165–169. 10.1016/s12467820(03)00035-1 12798851

